# Functional Polymorphisms in Dopaminergic Genes Modulate Neurobehavioral and Neurophysiological Consequences of Sleep Deprivation

**DOI:** 10.1038/srep45982

**Published:** 2017-04-10

**Authors:** Sebastian C. Holst, Thomas Müller, Amandine Valomon, Britta Seebauer, Wolfgang Berger, Hans-Peter Landolt

**Affiliations:** 1 Institute of Pharmacology and Toxicology, University of Zürich, Zürich, Switzerland; 2 Zurich Center of Interdisciplinary Sleep Research, University of Zürich, Zürich, Switzerland; 3 Zurich Center for Integrative Human Physiology, University of Zürich, Zürich, Switzerland; 4 Institute of Medical Molecular Genetics, University of Zürich, Schlieren, Switzerland; 5 Zurich Center of Neuroscience (ZNZ), University of Zurich and Federal Institute of Technology (ETH) Zurich, Zurich, Switzerland

## Abstract

Sleep deprivation impairs cognitive performance and reliably alters brain activation in wakefulness and sleep. Nevertheless, the molecular regulators of prolonged wakefulness remain poorly understood. Evidence from genetic, behavioral, pharmacologic and imaging studies suggest that dopaminergic signaling contributes to the behavioral and electroencephalographic (EEG) consequences of sleep loss, although direct human evidence thereof is missing. We tested whether dopamine neurotransmission regulate sustained attention and evolution of EEG power during prolonged wakefulness. Here, we studied the effects of functional genetic variation in the dopamine transporter (*DAT1*) and the dopamine D_2_ receptor (*DRD2*) genes, on psychomotor performance and standardized waking EEG oscillations during 40 hours of wakefulness in 64 to 82 healthy volunteers. Sleep deprivation consistently enhanced sleepiness, lapses of attention and the theta-to-alpha power ratio (TAR) in the waking EEG. Importantly, *DAT1* and *DRD2* genotypes distinctly modulated sleep loss-induced changes in subjective sleepiness, PVT lapses and TAR, according to inverted U-shaped relationships. Together, the data suggest that genetically determined differences in *DAT1* and *DRD2* expression modulate functional consequences of sleep deprivation, supporting the hypothesis that striato-thalamo-cortical dopaminergic pathways modulate the neurobehavioral and neurophysiological consequences of sleep loss in humans.

Sleep deprivation enhances sleepiness, impairs performance and alters electrical brain activity in a highly predictable fashion^1^,^2^,^3^,^4^. Modern lifestyle means that these sleep-loss related cognitive deficits have developed to a public health concern. In the laboratory, neurobehavioral deficits of sleep deprivation are accurately indexed by lapses of attention on the psychomotor vigilance task (PVT), which is considered a gold-standard measurement of sustained vigilant attention^3^,^5^. Neurophysiological consequences of sleep loss on the other hand, are reflected by distinct oscillations in the waking electroencephalogram (EEG)^6^,^7^. Importantly, these neurobehavioral and neurophysiological consequences vary widely among individuals. Ample evidence now demonstrates that genetic influences strongly modulate waking EEG oscillations and alters PVT lapses across prolonged waking, even within consecutive test sessions^8^,^9^,^10^,^11^,^12^. Consistent with a genetic contribution, the impact of sleep loss on distinct subjective, PVT, and neurophysiological markers of alertness, is trait-like and highly robust within individuals. Intriguingly, however, these variables don’t typically show a clear association with each other, but rather develop seemingly independently^13^,^14^. Here, we hypothesized that functional variation in genes impacting local dopaminergic neurotransmission affects sleep loss-induced changes in PVT lapses and the waking EEG along a similar trajectory.

The neurotransmitter dopamine contributes to the regulation of different brain functions, including sustained attention^15^ and EEG oscillations during wakefulness^16^,^17^. Wakefulness-promoting medications typically enhance dopaminergic neurotransmission and improve performance on the PVT during simulated night shifts and after sleep deprivation^18^,^19^,^20^. Nevertheless, a distinct role for dopamine in the proper regulation of sleep wake-states has long been controversial^21^. Intriguing recent data obtained with chemogenetic and optogenetic methods in flies and mice have started to change this view^22^,^23^,^24^. Moreover, genetically modified animals lacking a functional dopamine re-uptake transporter (DAT) exhibit prolonged wakefulness and shortened sleep^25^,^26^,^27^,^28^,^29^. In humans, a variable-number-tandem-repeat (VNTR) polymorphism (SNP-ID: rs28363170) of the gene encoding DAT (*SLC6A3* or *DAT1*) modulates individual effects of sleep loss on sleep rebound and sensitivity to caffeine, as well as rewards and punishments^30^,^31^. Homozygous ten-repeat allele (10R/10R) carriers of this polymorphism presumably have 15–20% reduced DAT protein expression compared to nine-repeat (9R) allele carriers^32^,^33^. Compared to the 9R carriers, 10R/10R homozygotes exhibits a clearly and more pronounced increase in sleep intensity after extended waking (as measured by EEG slow-wave activity [SWA] in non-rapid-eye-movement [NREM] sleep)^30^. This measure is the best established biomarker of increased sleep need after sleep loss^34^.

Dopamine D_2_ receptors were recently proposed to be an important part of the network that regulates sleep and wakefulness^35^,^36^. Nevertheless, the exact role of D_2_ receptor agonist and antagonists in modulating behavioral states remain controversial^37^. Alike DAT, these receptors are primarily expressed in the striatum. Common variants of the gene (*DRD2*) encoding the D_2_ receptor in humans influence reported habitual sleep duration^38^. Molecular imaging studies revealed that sleep deprivation reduces D_2_ receptor availability in the striatum^39^,^40^,^41^, and suggest that these receptors are involved in modulating visual attention in rested wakefulness and after sleep deprivation^42^. The *DRD2* gene includes a functional 957 C > T single nucleotide polymorphism (SNP-ID: rs6277) that affects levels and stability of mRNA and has been associated with 15–20% enhanced striatal D_2_ receptor availability in C-allele carriers compared to T-allele carriers^43^. Importantly, functional interactions between *DAT1* and *DRD2* genes^44^,^45^ appear to modulate dopamine-dependent neuronal activity according to an inverse U-shaped relationship^46^.

Based on the evidence presented above, we aimed at investigating the impacts of functional *DAT1* and *DRD2* polymorphisms on sleep deprivation-induced changes in subjective sleepiness, attentional lapses and the waking EEG in humans. We hypothesized that these genetic variants impact individual consequences of sleep loss and expected that they interact to modulate vigilant attention and EEG power during prolonged wakefulness according to U-shaped relationships.

## Results

### The impact of sleep deprivation on PVT performance and EEG oscillations are likely governed by separate mechanisms

**Figure 1:**
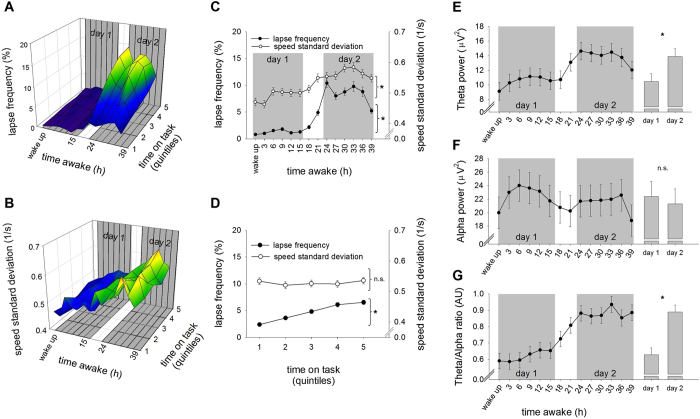
Psychomotor vigilance task (PVT) performance and waking EEG oscillations across prolonged wakefulness. Evolution of psychomotor vigilance task (PVT) performance and waking EEG oscillations across prolonged wakefulness. (**A**,**B**) Vertical axes (y-axes) on the 3D plots represent attention lapses (lapse frequency) and response variability (standard deviation of response speed), as a function of time awake (x-axis) and time-on-task (z-axis). Warmer colors represent higher lapse frequency and increased response variability. (**C**,**D**) 2D plots illustrating increased lapse frequency and response variability by sleep deprivation and increased lapse frequency by time-on-task. (**E**–**G**) Effects of sleep deprivation on waking EEG theta activity, alpha activity, and theta/alpha ratio (TAR). The gray background shows the data included in the statistical analyses, categorized as day 1 (baseline) and day 2 (sleep deprivation). Stars indicate least significant difference (LSD) between day 1 and day 2 (p < 0.0001).

To assess the consequences of sleep deprivation, performance on the PVT, subjective sleepiness and the waking EEG were quantified in 14 equally-spaced sessions across the 40 hours of prolonged wakefulness. From the baseline to the sleep deprivation day, PVT performance deteriorated (factor ‘*day*’: lapse frequency: 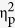 = 0.312; response variability: 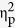 = 0.109), whereas the sleepiness score increased (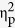 = 0.805; 2.43 ± 0.08 *vs*. 4.67 ± 0.09) (Fig. 1A–D; for detailed statistics, see Supplementary Table 1). The changes from sleep deprivation in lapse frequency and sleepiness revealed a tight association (r_s_^2^ = 0.110, p < 0.008, n = 64). Failed responses also increased with time on task within PVT sessions (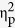 = 0.212), and this increase was amplified by sleep deprivation (‘*day*’ × ‘*time on task*’: 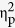 = 0.157). By contrast, response variability was virtually unaffected by time on task (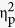 = 0.011). Significant interactions between ‘*day*’ and ‘*clock time*’ were observed for both neurobehavioral performance (lapse frequency: 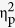 = 0.083; response variability: 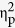 = 0.017) and sleepiness (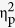 = 0.135), confirming that these state variables not only depend on sleep-wake history but also on diurnal fluctuations.

Because it was previously concluded that the behavioral consequences of sleep deprivation can dissociate from neurophysiological indices of elevated sleep pressure^47^, the evolution of electrical brain activity in five predefined frequency bands and the theta-to-alpha ratio (TAR) was quantified at 3-hour intervals across prolonged wakefulness. By determining the TAR, the signal to noise ratio (SNR) between-subjects (TAR: 1.76 ± 0.10) was enhanced compared to the theta (1.17 ± 0.20; t_13_ = −10.43, p < 0.0001) and alpha frequency bands (1.06 ± 0.08; t_13_ = −17.2, p < 0.0001), which improved the capability to detect genotype-dependent differences (see below). By contrast, within-subject SNR was only slightly reduced (TAR: 4.25 ± 2.13; theta: 4.55 ± 2.01; alpha: 4.83 ± 2.09; TAR vs. theta: t_80 _=_ _1.22, p > 0.22; TAR vs. alpha t_80_ = 2.11, p < 0.04).

Sleep deprivation increased theta activity (‘*day*’: 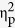 = 0.548) and TAR (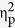 = 0.519), while alpha activity was unaffected (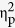 = 0.011, Fig. 1E–G, Supplementary Table 1). On the other hand, time of day affected theta (‘*clock time*’: 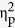 = 0.062; ‘*clock time*’ × ‘*day*’: 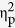 = 0.075) and alpha activity (‘*clock time*’: 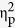 = 0.067; ‘*clock time*’ × ‘*day*’: 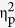 = 0.049), and also tended to modulate TAR (‘*clock time*’: 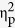 = 0.017; ‘*clock time*’ × ‘*day*’: 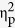 = 0.015). Consistent with the literature, the sleep deprivation induced changes in theta power and TAR were not associated with increased PVT lapse frequency (r_all_ < 0.076, p_all_ > 0.55), suggesting that these markers are governed by separate mechanisms.

### *DAT1* and *DRD2* genotypes affect PVT performance and subjective sleepiness

In the next step, the effects of the functional polymorphisms rs28363170 (‘*DAT1*’), rs6277 (‘*DRD2*’) and their combination (‘*DAT1-DRD2 combined*’) on the evolution of PVT performance during prolonged wakefulness were investigated. The p-values of all ANOVA’s investigating genetic effects were corrected for by multiple comparison using false discovery rate (FDR) correction (see Supplementary Table 2).

**Figure 2:**
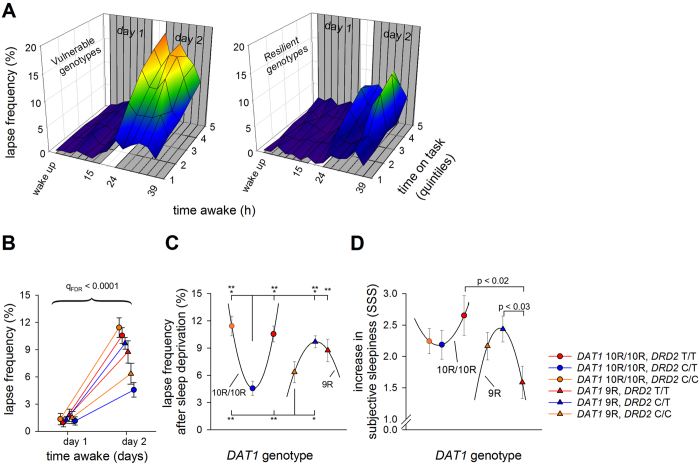
The neurobehavioral consequences of sleep deprivation split by the *DAT1-DRD2* combined genotypes. Effect of 40 hours sustained wakefulness on PVT lapses and subjective sleepiness (Stanford Sleepiness Scale [SSS]), split by the *DAT1-DRD2* combined genotypes. (**A**) Evolution of PVT lapses (y-axis) across time awake (x-axis) and time-on-task (z-axis) split into the most vulnerable *DAT1-DRD2* genotypes (10R/10R-C/C, 10R/10R-T/T, 9R-T/T and 9R-C/T; left) compared to the two more resilient genotypes (10R/10R-C/T and 9R-C/C; right). Warmer colors refers to higher lapse frequency. (**B**,**C**) Illustration of the highly significant interaction between the *DAT1-DRD2* genotypes and sleep deprivation for lapses of attention. (**B**) The evolution of lapses from baseline (day 1) to sleep deprivation (day 2). Significant differences between genotypes were only observed on day 2. (**C**): PVT lapse frequency on day 2 can be described by a U-shaped curve with an arbitrary horizontal axis split by the *DAT1-DRD2* combined genotypes. (**D**) Illustration of the interaction between the *DAT1-DRD2* genotypes and sleep deprivation (qFDR < 0.02) for subjective sleepiness (SSS). Similar to (**C**), the genotypes are plotted on a U-shaped curve, split by DAT1 genotype. Colors represent the six genetic groups (orange: *DRD2* C/C, blue: *DRD2* C/T, red: *DRD2* T/T, circles: *DAT1* 10R/10R, triangles: *DAT1* 9R) and are identical in panels B through D. Group sizes: *DAT1* 10R/10R, DRD2 C/C (lapses: n = 7, SSS: n = 9), *DAT1* 10R/10R, *DRD2* C/T (lapses: n = 14, SSS: n = 18), *DAT1* 10R/10R, *DRD2* T/T (lapses: n = 11, SSS: n = 13), *DAT1* 9R, DRD2 C/C (lapses: n = 6, SSS: n = 8), *DAT1* 9R, DRD2 C/T (lapses: n = 20, SSS: n = 24), *DAT1* 9R, DRD2 T/T (lapses: n = 6, SSS: n = 9). Significant post-hoc comparisons are illustrated by horizontal lines, whereas long vertical lines represent the genetic group investigated. Stars and p-values represent the least significant difference (LSD) following corresponding ANOVAs. Triple stars: p < 0.0001, double stars: p < 0.005, single stars: p < 0.02.

When PVT response speed was considered, complex interactions between the genotypes, sleep deprivation and diurnal rhythmicity were observed (‘*DAT1*’ × ‘*clock time*’ × ‘*day*’: 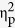 = 0.020; ‘*DAT1-DRD2 combined*’ × ‘*clock time*’ × ‘*day*’: 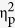 = 0.066). When investigating the sleep-deprivation induced change in PVT lapses, a significant modulation by the ‘*DRD2*’ genotype was observed, yet with a minuscule effect size (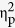 = 0.008). When ‘*DAT1*’ and ‘*DRD2*’ genotypes were combined, however, much larger effect sizes could be identified. The statistical analyses demonstrated a main effect of genotype (‘*DAT1-DRD2 combined*’: 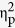 = 0.156), as well as interactions with time awake and clock time (‘*DAT1-DRD2 combined*’ × ‘*clock time*’: 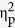 = 0.080; ‘*DAT1-DRD2 combined*’ × ‘*day*’: 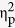 = 0.055; ‘*DAT1-DRD2*’ × ‘*clock time*’ × ‘*day*’: 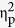 = 0.080) (Fig. 2 and Supplementary Figure 1). Post hoc testing revealed no significant differences at baseline (day 1), yet multiple significant effects on day 2 after sleep deprivation (Fig. 2B,C). These findings show that the 10R/10R-C/T and 9R-C/C genotypes had significantly fewer lapses after sleep deprivation than the remaining four *DAT1-DRD2* genotypes. Taken together, these results indicate that the *DAT1-DRD2* 10R/10R-C/T and 9R-C/C genotypes maybe more resilient to deteriorating psychomotor vigilance by sleep deprivation (Fig. 2A and Supplementary Figure 1). It also should be noted, that the U-shaped relationship observed on day 2 for PVT lapse frequency, closely mirrored the relative increase in PVT lapses after sleep deprivation (Supplementary Figure 1B).

Similar to PVT performance, the increase in subjective sleepiness by sleep deprivation was not modulated by *DAT1* and *DRD2* genotypes alone, yet by the combined *DAT1-DRD2* genotypes (‘*DAT1-DRD2 combined*’ × ‘*day*’: 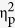 = 0.080). Intriguingly, the increase in subjective sleepiness resembled a similar U-shaped relationship, split by *DAT1* genotype (Fig. 2D).

### *DAT1* and *DRD2* genotypes affect the waking EEG during prolonged wakefulness

**Figure 3:**
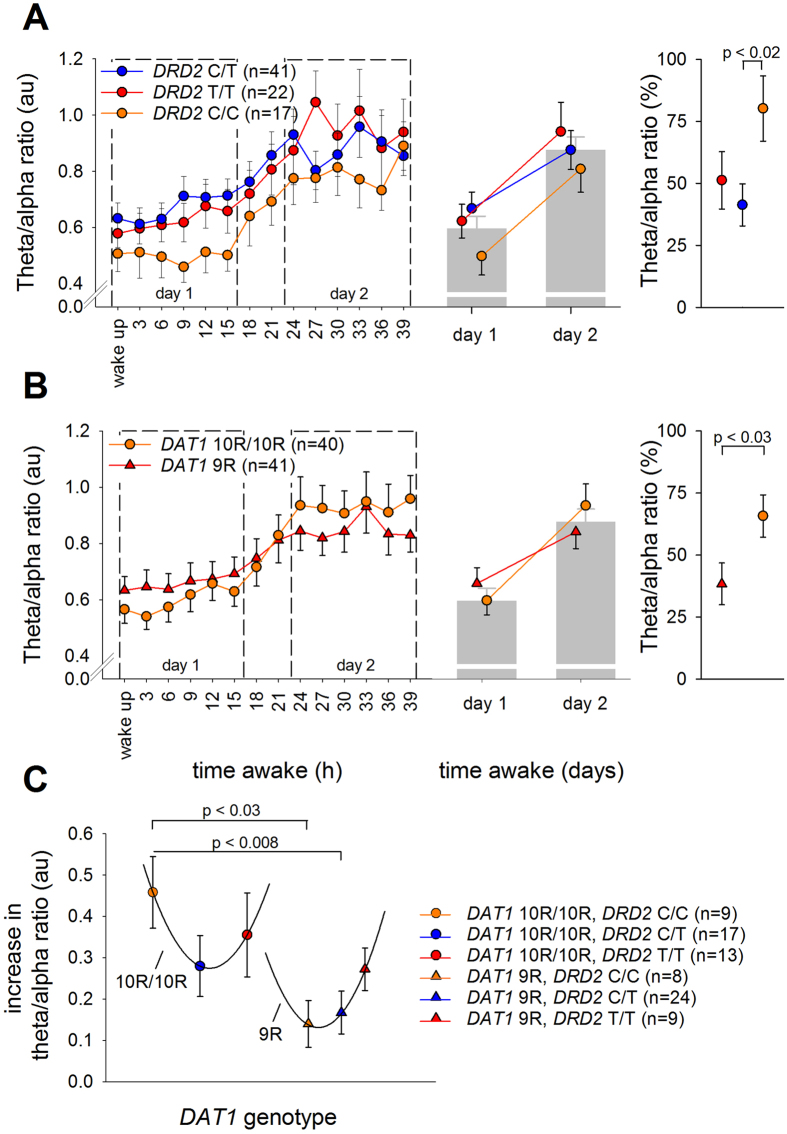
The neurophysiological consequences of sleep deprivation split by the *DAT1-DRD2* combined genotypes. Effect of 40 hours prolonged wakefulness on the increase in EEG theta-to-alpha ratio (TAR), split by *DAT1, DRD2*, and combined *DAT1-DRD2* genotypes, all showing significant genotype x sleep deprivation interactions (q_FDR_ < 0.02). The portion of the waking EEG recordings with eyes open was analyzed. (**A**,**B**) Left: TAR quantified at 3-hour intervals for *DAT1* (panel A; 10R/10R: orange, 9R: red) and *DRD2* (panel B; C/C: orange, C/T: blue, T/T: red) genotypes. Right: Change in TAR from day 1 to day 2 in *DAT1* and *DRD2* genotypes. Statistics revealed a significant effect of sleep deprivation on TAR, which was stronger in 10R/10R homozygotes than 9R-allele carriers of *DAT1* and in *DRD2* C/C homozygotes compared to C/T heterozygotes. (**C**) Increase in TAR from day 1 to day 2 in the combined *DAT1-DRD2* genotype groups. The sleep deprivation-induced increase in TAR is described by a U-shaped curve with an arbitrary horizontal axis, split by *DAT1* genotype. P-values represent the least significant difference (LSD) following the corresponding ANOVA.

Next, it was investigated whether the dopaminergic genotypes also influence waking EEG oscillations during prolonged wakefulness. Three-way ANOVAs revealed no effects of ‘*genotype*’, or their interaction, on the evolution of delta, theta, low-beta and high-beta frequency activity during prolonged waking (see Supplementary Table 2 for statistics). By contrast, *DAT1* (‘*DAT1*’ × ‘*day*’: 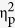 = 0.059) and *DAT1-DRD2* combined genotypes (‘*DAT1-DRD2 combined*’ × ‘*day*’: 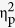 = 0.109) affected the change in alpha activity by sleep deprivation. Similarly, *DAT1* (‘*DAT1*’ × ‘*day*’: 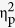 = 0.049), *DRD2* (‘*DRD2*’ × ‘*day*’: 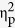 = 0.071) and *DAT1-DRD2* combined genotypes (‘*DAT1-DRD2 combined*’ × ‘*day*’: 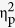 = 0.137) modulated TAR (Fig. 3). Specifically, the percentage increase in TAR after sleep deprivation was blunted in *DAT1* 9R-allele carriers when compared to *DAT1* 10R/10R homozygotes (Fig. 3A) and in *DRD2* C/T heterozygotes compared to C/C homozygotes (Fig. 3B).

Similar to PVT lapses, the increase in TAR after sleep deprivation may be described by a U-shaped relationship, split by *DAT1* genotype (Fig. 3C). Intriguingly, however, whereas the curve associated with *DAT1* 10R/10R was reminiscent of the sleep deprivation-induced consequences on sleepiness and neurobehavioral performance, the curve associated with the *DAT1* 9R genotype appeared inverted.

## Discussion

Modulation of dopaminergic neurotransmission in Drosophila and mouse mutants are associated with profound modulations of wakefulness and sleep^26^,^27^,^28^,^29^. Recent chemogenetic and optogenetic studies in flies and mice confirmed this crucial role of dopamine in regulating sleep-wake behaviors^22^,^23^,^24^. Nevertheless, the contribution of the dopaminergic system to sleep-wake regulation in humans is still only poorly described. The present study revealed that functional polymorphisms of *DAT1* and *DRD2* together modulate the neurobehavioral, subjective and neurophysiological consequences of sleep deprivation. Nevertheless, the distinct contributions of *DAT1* and *DRD2* genotypes to wakefulness-induced changes in physiological fatigue and sustained attention on one hand^48^,^49^, and EEG-derived markers of vigilance on the other hand^6^,^7^,^50^ appear to differ.

The relationships between dopaminergic neurotransmission and brain functions are complex. They are task specific and depend on the different dopamine receptor families and dopaminergic pathways involved^51^,^52^. Furthermore, epistasis between genetic variants of *DAT1* and *DRD2* genes has been described^44^. Functional magnetic resonance imaging data suggested that activation of the striatum in a monetary reward paradigm depends on genetic variants of both *DAT1* and *DRD2* genes. Indeed, Li *et al*. showed in more than 1200 healthy adults of European decent that individuals with elevated levels of dopaminergic transmission, associated with *DAT1* 9R and *DRD2* C/C genotypes, showed better performance on a serial memory task than individuals with lower dopaminergic transmission^45^. Similarly, activation of the prefrontal cortex during a working memory and recognition task could be described by a U-shaped relationship depending on functional variants of *DAT1* and *DR2D* genes modifying dopaminergic neurotransmission^46^. Consistent with these data, we found that increased lapse frequency on the PVT, subjective sleepiness, and EEG TAR after sleep deprivation can be described by U-shaped curves split by *DAT1* genotype. The curves reflecting PVT performance and subjective sleepiness were similar, suggesting that the observed effects may reflect inherent consequences of sleep deprivation. This is intriguing, especially when considering that the sample sizes differed by 20% (between 64 and 80 individuals) for PVT recordings and subjective sleepiness ratings. Considering PVT lapse frequency, our data suggests that subjects carrying the *DAT1-DRD2* 10R/10R-C/T or the *DAT1-DRD2* 9R-C/T genotypes, are more resilient to sleep deprivation than the remaining four genotypes. When inspecting the increase in subjective sleepiness, however, the *DAT1-DRD2* 9R-C/C genotype is significantly less affected by prolonged wakefulness than the *DAT1-DRD2* 10R/10R-T/T and 9R-C/T genotypes. These effects could not be attributed to either the *DAT1* or the *DRD2* genotypes alone, and show that genotype dependent discrepancies between subjective and objective markers of sleep loss are present.

The statistical analyses revealed that the combined effects of *DAT1* and *DRD2* genotypes explained about 8% of the observed variability in subjective sleepiness, 15% in PVT lapse frequency, and 13% in EEG TAR. The magnitude of these effect sizes can be considered moderate to large^53^,^54^. They appear considerably larger than what was previously reported for other genetic variants^11^, which emphasizes the robustness of the findings. The direction by which the *DAT1* and *DRD2* genotypes modulated sustained attention and subjective sleepiness after sleep deprivation should also be noted (Fig. 2). Interestingly, the effect of the *DRD2* genotypes was almost inverted in 10R/10R homozygotes and 9R allele carriers of *DAT1*. The different relationships highlight the importance of considering related signaling pathway components, rather than single genetic variants, when examining possible genetic influences on the vulnerability to sleep deprivation. Future studies may investigate the relative quantitative contributions of DAT and D_2_ protein expression on neurobehavioral and neurophysiological consequences of sleep deprivation.

Not only the neurobehavioral consequences of sleep deprivation, but also those of ‘time-on-task’ in rested and sleep deprived subjects were associated with common genetic variants^55^. Lim and colleagues investigated the impact of six polymorphisms related to dopaminergic neurotransmission on time-on-task-dependent decrements in PVT performance in 350 adults in the absence of sleep deprivation^12^. The authors concluded that the VNTR in *DAT1*, but not the ANKK1 polymorphism of *DRD2* (SNP-id: rs1800497), modulated the decline in reaction speed associated with increasing task duration. Although both lapse frequency and response variability increased with time-on-task, our repeated measure analyses did not corroborate a *DAT1* genotype-dependent modulation. Further studies are needed to explore whether the discrepancy reflects differences in study design or reduced power because of the smaller sample size in the present study.

Previous work stressed the lack of clear associations between PVT performance, sleepiness and neurophysiological markers of vigilance^13^,^14^,^47^,^56^. Our analyses corroborate these discrepancies, but only when the 9R-allele carriers of the *DAT1* gene are considered. In 10R/10R homozygotes, the suggested U-shaped relationships between *DRD2* genotypes and increased lapse frequency, subjective sleepiness, and EEG TAR after sleep deprivation were remarkably similar. Given the lack of association between increased lapse frequency and theta activity or TAR, however, the genotypes may independently affect behavioral and EEG measures. This implies that they are likely governed by separate mechanisms, which are influenced by dopaminergic neurotransmission.

The present study indicates that TAR may be a promising novel physiological marker of reduced vigilance associated with sleep deprivation. Indeed, work investigating the action of adrenergic and dopaminergic compounds on the multiple sleep latency test suggested that changes in TAR may predict vigilance^6^. In addition, based on comparisons of 85 electrophysiological EEG measures in 20 healthy adults it was concluded that the TAR is the best EEG marker to predict changes in vigilance from wakefulness to sleep, and from wakefulness to superficial stage 1 sleep^50^. As reported here, the TAR enhanced between-subjects signal-to-noise-ratio when compared to either alpha or theta activity alone. Furthermore, the TAR showed a robust increase with sleep deprivation. Especially the impact of *DAT1* genotype on increased TAR after sleep deprivation should be highlighted. Previous findings of our group revealed that homozygous 10R/10R carriers exhibited a more pronounced increase than 9R-allele carriers in nocturnal slow wave sleep and EEG slow-wave activity after prolonged wakefulness, suggesting that 10R/10R homozygotes show a more pronounced homeostatic response to sleep loss^30^. The data presented here support this notion and show that 10R/10R homozygotes of *DAT1* display a stronger increase in TAR by sleep deprivation. Moreover, the data also reveal that *DRD2* C/C homozygotes show a stronger increase in TAR after sleep-loss than the C/T heterozygotes, which suggests that also the *DRD2* polymorphism is involved in homeostatic sleep-wake regulation. Nocturnal sleep EEG recordings before and after sleep deprivation are needed to further corroborate these findings.

The DAT and dopamine D_2_ receptors are primarily expressed in the striatum. This brain region may be an important part of the intrinsic system that controls sleep and wakefulness^35^,^36^. The present genetic findings support data collected in animals^24^,^25^,^26^,^27^,^28^,^57^ and observations from brain imaging^30^,^39^,^40^,^42^ and epidemiological^38^ studies in humans, suggesting prominent roles for DAT and D_2_ receptors in sleep-wake regulation. They highlight the importance of the striatum in regulating changes in vigilant attention, subjective sleepiness, and neurophysiological EEG oscillations during prolonged wakefulness. It is likely that striatal dopaminergic signaling, together with the prefrontal cortex and related brain neurotransmitter- and neuromodulator- systems, contributes to the control of the sleep-wake continuum^58^. The data presented here suggests that common functional genetic variants linked to altered striatal dopaminergic neurotransmission contribute to individual neurobehavioral consequences of sleep loss. These genetic variants could help define trait-like risks for sleep deprivation-related motor vehicle- and work-related accidents. Moreover, the genetic variants may help explain individual differences in the efficacy of dopaminergic psychostimulants, used to mitigate the detrimental effects of sleep-wake disorders and sleep deprivation.

## Methods

### Study participants and sleep deprivation protocol

Eighty-two healthy right-handed volunteers (12 females) between 19 and 35 years completed the sleep deprivation experiment (see refs 30,59, for further details on the study protocols). All volunteers reported to be good sleepers, adhere to regular bedtimes, be in good physical health, and have no history of neurological or psychiatric disorders. Two months prior to enrollment, subjects did not consume any medication or illicit drugs, did not pass through time zones and consumed only moderate amounts of caffeine and alcohol. Three participants were moderate smokers (<10 cigarettes per day). Good sleep quality (no undiagnosed sleep disorders) and efficiency (>85%) were confirmed in a screening night in the sleep laboratory prior to study inclusion. At least three days before study initiation, participants were instructed to wear a wrist activity monitor on their non-dominant arm, fill in a sleep-wake diary, refrain from caffeine and alcohol, and strictly maintain 8 hours’ time in bed, corresponding to the scheduled bedtimes of the study.

All participants completed 40 hours of constantly supervised wakefulness, preceded by two consecutive 8-hour sleep episodes in the sleep laboratory (adaptation and baseline nights). During prolonged waking, the consequences of sleep loss were monitored at 3 hour intervals with standardized test sessions, including subjective sleepiness ratings, PVT and waking EEG recordings.

The study protocols were approved by the ethics committee of the Canton of Zürich for research on human subjects. Written informed consent was obtained from all participants prior to the experiments, as required according to the principles in the Declaration of Helsinki. All subjects received financial compensation for their participation.

### Genotyping

Genomic DNA was extracted from 3 ml fresh EDTA-blood (Wizard^®^ Genomic DNA Purification Kit, Promega, Madison, WI). The rs28363170 polymorphism of *DAT1* was determined by allele-specific PCR on an MJ Research PTC-225 thermal cycler (MJ Research/Bio-Rad, Reno, NV) at an annealing temperature of 67 °C. Forward primer, 5′-tgtggtgtagggaacggcctga-3′, and reverse primer, 5′-cttcctggaggtcacggctcaa-3′, with HOT FIREPol^®^ DNA Polymerase was used. The 430–480 bp PCR products were analyzed by agarose gel electrophoresis. The functional polymorphisms rs6277 of *DRD2* was determined using a Taqman^®^ SNP Genotyping Assay (Life Technologies Europe B.V.; probe number: C__11339240_10). Allelic discrimination analysis was performed with SDS v2.2.2 software (Applied Biosystems, Foster City, CA, USA). All genotypes were replicated at least once for independent confirmation of results. The *DRD2* genotype of one individual is lacking due to missing genetic material. Comparisons of demographic variables between *DAT1* and *DRD2* genotypes included weight, height, body mass index (BMI), age, gender, habitual alcohol and caffeine consumption, self-reported daily sleep duration, daytime sleepiness and trait anxiety (Table 1). No variable revealed a difference between the genotypes. Fishers exact test revealed that the *DRD2* and *DAT1* genetic groups were similarly distributed, both in the full (n = 81, p > 0.39) and in the subsample (n = 64; p > 0.30).

### Psychomotor vigilance task (PVT) and subjective sleepiness

Vigilant attention during sleep deprivation was assessed by a PC-implemented e-Prime version (Psychology Software Tools Inc., Pittsburgh, PA) of the psychomotor vigilance task^60^. Eighteen individuals were excluded from the analyses because they performed a different, non-computerized version of the task, resulting in a sample size of 64 subjects for the PVT task analyses.

The PVT is a simple reaction time task. When a digital millisecond counter starts to scroll in the center of the computer screen, subjects have to press a button with their right index finger on a response box (standardized keyboard) connected to the PC. Subjects received oral instructions and performed one training session on the evening prior to the baseline night. For each PVT trial, 100 stimuli were presented (random inter-stimulus intervals: 2–10 s). To assess performance as a function of ‘time on task’ (TOT), the task was divided into five quintiles of 20 trials. Two extensively validated PVT variables were quantified^48^,^56^,^61^,^62^: ‘lapses of attention’ (defined as the number of trials with reaction times longer than 500 ms) and ‘standard deviation of response speed’ (calculated based on inverse reaction times and referred to as response variability). Both these variables are prominent markers of sleep loss and sensitive to TOT.

Immediately prior to all PVT assessments, a validated German version of the Stanford Sleepiness Scale was administered^63^. The sleepiness ratings of all 82 subjects were included in the analyses.

### Waking EEG recordings

At 3-hour intervals throughout prolonged wakefulness, standardized waking EEG recordings with polygraphic PSA24 (Braintronics Inc., Almere, The Netherlands) (n = 18; see: ref. 2) or Artisan (Micromed, Mogliano Veneto, Italy) (n = 64; see e.g.: ref. 64) amplifiers were performed. The EEG data recorded with PSA24 amplifiers, were analogue band-pass filtered (−3 dB at 0.16 Hz; −3 dB at 102 Hz) and sampled at 512 Hz, then digitally low-pass filtered (−3 dB at 49 Hz) and stored with a resolution of 128 Hz. The EEG data recorded with Artisan amplifiers, were analogue band-pass filtered (−3 dB at 0.15 Hz; −3 dB at 67.2 Hz), sampled, and stored with a resolution of 256 Hz. Note that *DAT1* and *DRD2* genotypes were similarly distributed between PSA24 and Artisan amplifiers (p_all_ > 0.87, Fishers exact test). The standard EEG derivation for sleep state scoring, C3M2, was analyzed.

At least 1 hour before each recording, subjects were confined to the sleep-laboratory (constant temperature: 19–21 °C; light intensity: <150 lux). Fifteen minutes before each recording, subjects stayed in their own private bedroom. The recordings consisted of an initial 3-min recording period with eyes closed, followed by a 5-min period with eyes open, while fixating a black dot on the wall. During all recordings, study participants comfortably relaxed in a chair, and placed their chin on an individually adjusted chinrest. Artifacts were visually identified and excluded. Due to a technical problem, the data of one subject needed to be excluded. The power spectra of artifact-free, 2-s EEG epochs (50% overlap, Hanning window, frequency resolution 0.5 Hz) recorded with eyes open were computed and analyzed between 0–30 Hz.

To investigate changes in the waking EEG across prolonged time awake, spectral power in the standard EEG bands, delta (1–4.5 Hz), theta (5–7.5 Hz), alpha (8–11.5 Hz), low-beta (12–19.5 Hz), and high-beta (20–30 Hz) was computed. Previous research has shown that the wakefulness-induced changes are particularly pronounced in theta (~4–8 Hz) and alpha (~8–12 Hz) ranges, as well as in the theta-to-alpha ratio (TAR)^6^,^7^,^50^. The EEG power and ratio values were transformed by a base 10 logarithm, to approximate a Gaussian distribution.

### Statistical analyses

Spearman’s rank-correlation coefficients (r_s_), as well as two-, three- and four-way mixed-model ANOVAs (analyses of variance) for repeated measures were computed. The covariance matrix applied was first order autoregressive and subjects were considered as random effect. To estimate the influences of circadian and homeostatic sleep regulatory processes^34^, the effect of time awake was analyzed by considering the within-subjects’ factors ‘*clock time*’ (8:00, 11:00, 14:00, 17:00, 20:00, 23:00) and ‘*day*’ (baseline *vs*. sleep deprivation day). In 22 participants, the assessments at 17:00 and 20:00 were missing due to neuroimaging^59^. For the analyses of PVT performance, an additional factor ‘*time on task (TOT*)’ (quintiles 1–5) was added. Within each quintile, lapse frequency was defined as the percentage of lapse trials and response variability as the standard deviation of the mean response time. The ANOVAs testing overall effects of sleep loss independently of genotype are shown in Supplementary Table 1. To assess genetic effects, separate ANOVAs with the following genotypes were performed: ‘*DAT1*’ (9R; 10R/10R); ‘*DRD2*’ (C/C; C/T; T/T); ‘*DAT1-DRD2 combined*’ (9R-C/C; 9R-C/T; 9R-T/T; 10R/10R-C/C; 10R/10R-C/T; 10R/10R-T/T). Effect sizes (partial eta squared: 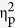) were calculated from corresponding ANOVA F-values and degrees of freedom. Effect sizes of 0.0099, 0.0588 and 0.1379 are considered small, moderate and large, respectively^53^,^54^. To correct for multiple comparisons, p-values originating from ANOVAs investigating genetic effects were corrected by false discovery rate (FDR) correction^65^. Corrected p-values (q_FDR_-values) below 0.05 were considered significant; post-hoc analyses were only performed when the respective main effect and/or interactions of the ANOVA withstood FDR correction. Results of all FDR corrected ANOVAs containing the factor ‘*genotype*’ are listed in Supplementary Table 2. Unless otherwise specified, only significant effects and interactions are mentioned. Following FDR corrected significant ANOVAs, least significant difference (LSD) post-hoc comparisons were performed.

Signal-to-noise ratios (SNR) were defined as group means (μ) divided by the standard deviation (σ) of EEG power in a given frequency band:


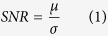


Following this classification, between-subject SNR was defined as the EEG band-power mean and standard deviation per session across the 81 subjects:


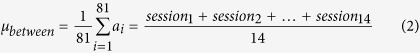



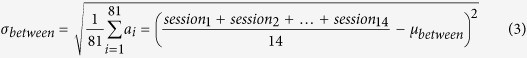


On the other hand, within-subject SNR was defined as the EEG band-power mean and standard deviation per subject across the 14 sessions:


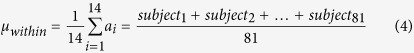



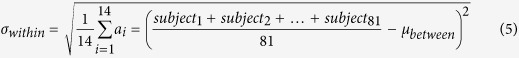


Signal to noise ratios across EEG bands, were compared using paired student t-tests.

Statistical analyses were performed with SAS 9.4 software (SAS Institute, Cary, NC), whereas PVT performance was assessed using IBM SPSS Statistics 22 (IBM Corp., Armonk, USA). Throughout, estimated means and standard errors of the respective ANOVAs are presented. For illustrative purposes, genetically-determined higher dopaminergic signaling (*DAT1* 10R/10R and *DRD2* C/C genotypes) was illustrated in orange color, intermediate dopaminergic signaling (*DRD2* C/T genotype) in blue color, and lower dopaminergic signaling (*DAT1* 9R and *DRD2* T/T genotypes) in red color when appropriate.

## Additional Information

**How to cite this article:** Holst, S. C. *et al*. Functional Polymorphisms in Dopaminergic Genes Modulate Neurobehavioral and Neurophysiological Consequences of Sleep Deprivation. *Sci. Rep.* **7**, 45982; doi: 10.1038/srep45982 (2017).

**Publisher's note:** Springer Nature remains neutral with regard to jurisdictional claims in published maps and institutional affiliations.

## Supplementary Material

Supplementary Information

## Figures and Tables

**Table 1:** Demographic characteristics of study participants.

	*DRD2 genotypes*		F_2,77_ (p)	*DAT1 genotypes*		F_2,79_ (p)
Weight (kg)	T/T	70.4 ± 1.81	0.01 (0.99)	9R	69.4 ± 1.48	0.63 (0.42)
	C/T	70.6 ± 1.32		10R/10R	73.7 ± 1.74	
	C/C	70.4 ± 2.05				
Height (cm)	T/T	178.6 ± 1.54	0.42 (0.66)	9R	177.9 ± 1.12	0.08 (0.78)
	C/T	177.0 ± 1.13		10R/10R	177.4 ± 1.14	
	C/C	178.2 ± 1.75				
BMI (kg/m^2^)	T/T	22.0 ± 0.39	0.50 (0.69)	9R	22.1 ± 0.29	1.98 (0.16)
	C/T	22.5 ± 0.27		10R/10R	22.6 ± 0.29	
	C/C	22.1 ± 0.42				
Age (years)	T/T	23.5 ± 0.65	0.97 (0.39)	9R	24.3 ± 0.48	0.07 (0.79)
	C/T	24.3 ± 0.48		10R/10R	24.1 ± 0.48	
	C/C	24.8 ± 0.74				
Alcohol consumption (drinks/week)	T/T	3.39 ± 0.61	0.48 (0.63)	9R	3.00 ± 0.44	0.23 (0.63)
	C/T	3.27 ± 0.45		10R/10R	3.30 ± 0.45	
	C/C	2.56 ± 0.69				
(mg/day)	T/T	119.1 ± 28.07	0.45 (0.65)	9R	114.7 ± 20.46	0.81 (0.37)
	C/T	138.5 ± 20.56		10R/10R	140.9 ± 20.72	
	C/C	104.2 ± 31.93				
Habitual sleep duration (hours)	T/T	7.15 ± 0.15	1.49 (0.24)	9R	7.31 ± 0.11	1.11 (0.29)
	C/T	7.36 ± 0.11		10R/10R	7.14 ± 0.11	
	C/C	7.03 ± 0.17				
Daytime Sleepiness (ESS)	T/T	7.32 ± 0.59	1.91 (0.16)	9R	7.31 ± 0.50	0.70 (0.40)
	C/T	7.46 ± 0.43		10R/10R	7.04 ± 0.59	
	C/C	5.94 ± 0.67				
Trait Anxiety (TAI)	T/T	34.0 ± 1.65	0.58 (0.57)	9R	35.5 ± 1.39	0.30 (0.58)
	C/T	35.9 ± 1.21		10R/10R	35.0 ± 1.64	
	C/C	36.48 ± 1.88				
	***DRD2 genotypes***		**(p)**	***DAT1 genotypes***		**(p)**
Gender ratio (% females)	T/T	13.6	(1.00)	9R	12.2	(0.75)
	C/T	14.6		10R/10R	15.4	
	C/C	11.8				

Demographic variables compared between *DRD2* and *DAT1* genotypes based on validated self-report questionnaires. Data represent means ± SEM; Statistics originate from one-way ANOVAs and Fisher’s exact test (gender ratio only). Caffeine consumption per serving was calculated based on self-reported values^64^,^66^. Similarly, as reported in the table, no significant difference between genotypes were observed in the subsample of 64 individuals (data not shown). TAI: Trait Anxiety Inventory^67^; ESS: Epworth Sleepiness Scale^68^.
