# Supplementary material for: Functional Polymorphisms in Dopaminergic Genes Modulate Neurobehavioral and Neurophysiological Consequences of Sleep Deprivation
Source: Sci Rep. 2017 Apr 10;7:45982. doi: 10.1038/srep45982 (PMC5385564; doi:10.1038/srep45982)
Supplement: Supplementary Information [file srep45982-s1.pdf]

FUNCTIONAL POLYMORPHISMS IN DOPAMINERGIC GENES MODULATE NEUROBEHAVIORAL AND NEUROPHYSIOLOGICAL CONSEQUENCES OF SLEEP DEPRIVATION

Supplementary information

Sebastian C. Holst[1,2,3], Thomas Müller[1], Amandine Valomon[1,2,3], Britta Seebauer[4,5], Wolfgang Berger[3,4,5] and Hans-Peter Landolt[1,2,3]

[1] Institute of Pharmacology and Toxicology, University of Zürich, Zürich, Switzerland
[2] Zurich Center of Interdisciplinary Sleep Research, University of Zürich, Zürich, Switzerland
[3] Zurich Center for Integrative Human Physiology, University of Zürich, Zürich, Switzerland
[4] Institute of Medical Molecular Genetics, University of Zürich, Schlieren, Switzerland
[5] Zurich Center of Neuroscience (ZNZ), University of Zurich and Federal Institute of Technology (ETH) Zurich, Zurich, Switzerland

Abbreviated title: *Dopaminergic polymorphisms and human sleep loss*

Submitted to: **Scientific Reports**
February 20, 2017

Supplementary figure 1.

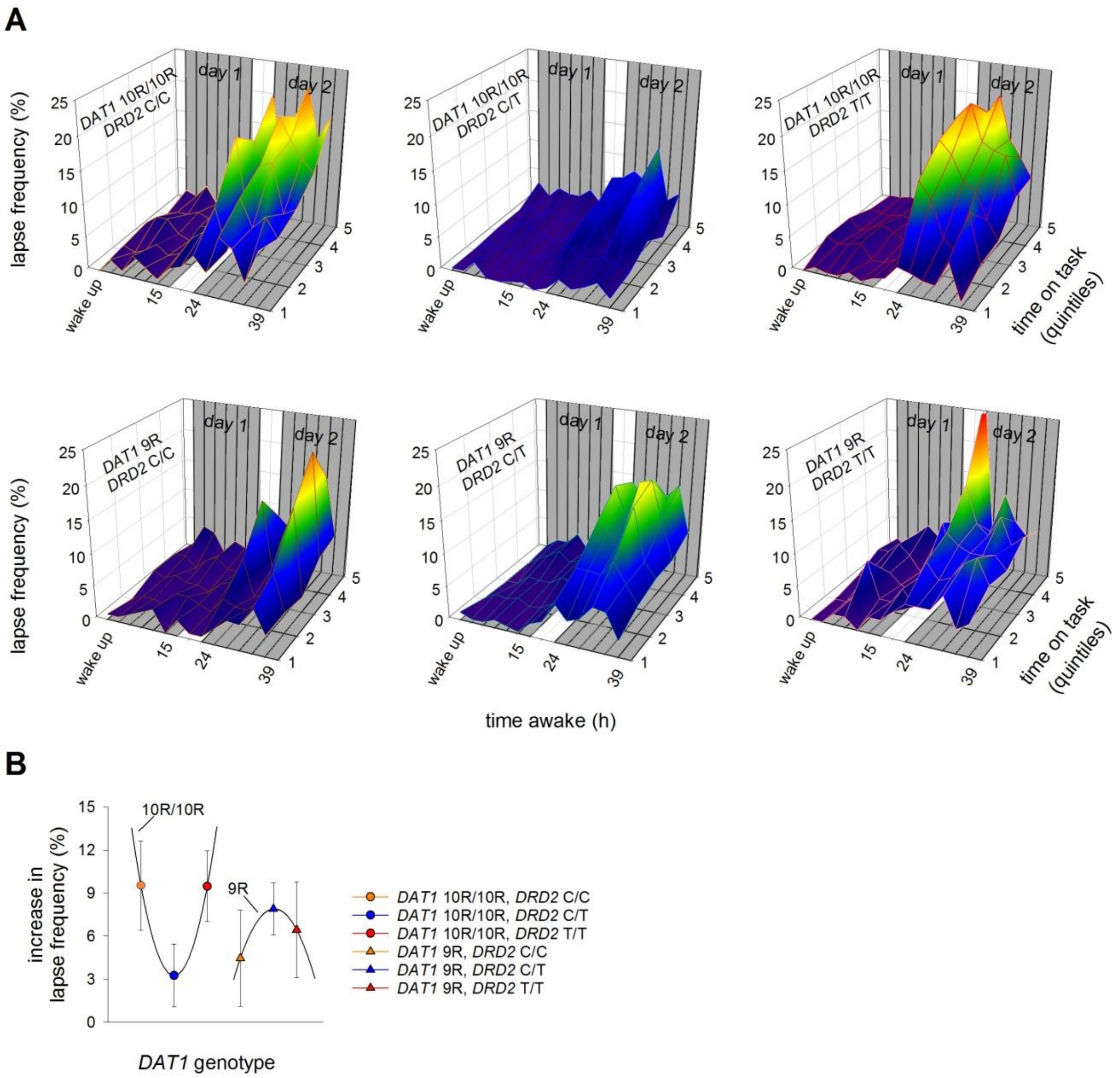

Illustration of the PVT lapse frequency data included in statistical models. **A:** PVT attention lapses (y-axis) as a function of time-on-task (z-axis) and time awake (x-axis). Warmer colors refers to higher lapse frequency. B: The relative increase in PVT lapse frequency from baseline to the sleep deprived day split by the six *DAT1-DRD2* genotypes plotted on a U-shaped curve. The figure resembles the reported increase in subjective sleepiness (Fig. 2C).

Supplementary table 1. Results of all genotype independent ANOVAs.

		F-value	p-value
PVT lapses	Session	$F_{5,544}$ = 9.41	$1.31E^{-08}$
	ToT	$F_{4,520}$ = 34.92	$7.68E^{-26}$
	Day	$F_{1,1183}$ = 536.60	$3.44E^{-98}$
	Session x ToT	$F_{20,279}$ = 0.70	0.83
	Session x Day	$F_{5,544}$ = 9.87	$4.82E^{-09}$
	ToT x Day	$F_{4,520}$ = 24.19	$2.26E^{-18}$
	Session x ToT x Day	$F_{20,279}$ = 1.16	0.29
PVT standard deviation	Session	$F_{5,734}$ = 6.89	$2.70E^{-06}$
	ToT	$F_{4,845}$ = 2.31	$5.61E^{-02}$
	Day	$F_{1,2122}$ = 259.21	$4.10E^{-55}$
	Session x ToT	$F_{20,390}$ =0.81	0.71
	Session x Day	$F_{5,734}$ = 2.50	$2.96E^{-02}$
	ToT x Day	$F_{4,845}$ = 0.87	0.48
	Session x ToT x Day	$F_{20,390}$ = 1.12	0.33
Subjective sleepiness	Session	$F_{5,567}$ = 17.73	$2.44E^{-16}$
	Day	$F_{1,218}$ = 920.00	$3.58E^{-80}$
	Session x Day	$F_{5,555}$ = 3.41	$4.85E^{-03}$
Waking EEG – Delta band 1 - 4.5 Hz	Session	$F_{5,619}$ = 7.02	$2.17E^{-06}$
	Day	$F_{1,213}$ = 108.81	$7.67E^{-21}$
	Session x Day	$F_{5,573}$ = 4.99	$1.75E^{-04}$
Waking EEG – Theta band 5 – 7.5 Hz	Session	$F_{5,626}$ = 8.26	$1.44E^{-07}$
	Day	$F_{1,193}$ = 233.57	$4.56E^{-35}$
	Session x Day	$F_{5,566}$ = 9.14	$2.22E^{-08}$
Waking EEG – Alpha band 8 – 11.5 Hz	Session	$F_{5,649}$ = 9.33	$1.36E^{-08}$
	Day	$F_{1,169}$ = 1.81	0.18
	Session x Day	$F_{5,569}$ = 5.87	$2.69E^{-05}$
Waking EEG – Theta/Alpha ratio	Session	$F_{5,636}$ = 2.14	$5.88E^{-02}$
	Day	$F_{1,164}$ = 177.14	$6.81E^{-28}$
	Session x Day	$F_{5,556}$ = 1.71	0.13
Waking EEG – low Beta band 12-19.5 Hz	Session	$F_{5,598}$ = 13.65	$1.24E^{-12}$
	Day	$F_{1,207}$ = 24.62	$1.45E^{-06}$
	Session x Day	$F_{5,556}$ = 20.72	$5.27E^{-19}$
Waking EEG – high Beta band 20-30 Hz	Session	$F_{5,590}$ = 3.46	$4.34E^{-03}$
	Day	$F_{1,182}$ = 5.66	$1.84E^{-02}$
	Session x Day	$F_{5,537}$ = 6.77	$3.91E^{-06}$

Subscript F values represent numerator and denominator degrees of freedom, respectively. Red values are below the standard 5% alpha criteria.

Supplementary table 2. Overview and results of all genotype related ANOVAs performed.

		DAT1			***DRD2***			***DAT1-DRD2***		
		F-value	p-value	q-value	F-value	p-value	q-value	F-value	p-value	q-value
**PVT lapses**	Genotype	$F_{1,134}$ = 1.00	0.32	0.57	$F_{2,135}$ = 2.20	0.14	0.24	$F_{5,128}$ = 4.75	$5.14E^{-4}$	$1.82E^{-3}$
	Session	$F_{5,533}$ = 9.32	$1.61E^{-08}$	$1.06E^{-07}$	$F_{5,508}$ = 7.61	$6.64E^{-07}$	$3.74E^{-06}$	$F_{5,509}$ = 6.59	$5.84E^{-6}$	$2.61E^{-5}$
	ToT	$F_{4,509}$ = 34.44	$1.86E^{-25}$	$2.75E^{-24}$	$F_{4,503}$ = 30.79	$6.01E^{-23}$	$8.38E^{-22}$	$F_{4,451}$ = 25.95	$2.22E^{-19}$	$2.75E^{-18}$
	Day	$F_{1,1154}$ = 530.44	$7.17E^{-97}$	$1.70E^{-94}$	$F_{1,1141}$ = 502.83	$1.46E^{-92}$	$1.73E^{-90}$	$F_{1,1024}$ = 432.91	$1.82E^{-80}$	$1.44E^{-78}$
	Genotype x Session	$F_{5,533}$ = 0.48	0.79	0.91	$F_{10,510}$ = 2.10	$2.32E^{-02}$	$6.10E^{-02}$	$F_{25,531}$ = 1.85	$7.54E^{-3}$	$2.29E^{-2}$
	Genotype x ToT	$F_{4,509}$ = 0.23	0.92	0.98	$F_{8,500}$ = 0.65	0.73	0.91	$F_{20,452}$ = 0.76	0.76	0.91
	Genotype x Day	$F_{1,1154}$ = 0.57	0.45	0.71	$F_{2,1135}$ = 4.82	$8.23E^{-03}$	$2.44E^{-02}$	$F_{1,1023}$ = 12.01	$2.55E^{-11}$	$1.95E^{-10}$
	Session x ToT	$F_{20,271}$ = 0.69	0.84	0.94	$F_{20,265}$ = 0.77	0.75	0.91	$F_{20,254}$ = 0.76	0.76	0.91
	Session x Day	$F_{5,533}$ = 9.76	$6.17E^{-09}$	$4.30E^{-08}$	$F_{5,508}$ = 7.60	$6.79E^{-07}$	$3.74E^{-06}$	$F_{5,509}$ = 6.27	$1.17E^{-5}$	$5.03E^{-5}$
	ToT x Day	$F_{4,509}$ = 23.84	$4.31E^{-18}$	$4.44E^{-17}$	$F_{4,503}$ = 23.60	$6.66E^{-18}$	$6.58E^{-17}$	$F_{4,451}$ = 19.04	$1.75E^{-14}$	$1.49E^{-13}$
	Genotype x ToT x Day	$F_{4,509}$ = 0.42	0.80	0.91	$F_{8,500}$ = 0.59	0.79	0.91	$F_{20,452}$ = 0.87	0.63	0.86
	Genotype x Session x Day	$F_{5,533}$ = 0.32	0.90	0.97	$F_{10,510}$ = 2.06	$2.59E^{-02}$	$6.74E^{-02}$	$F_{25,531}$ = 1.85	$7.75E^{-3}$	$2.33E^{-2}$
	Genotype x Session x ToT	$F_{20,271}$ = 0.32	1.00	1.00	$F_{40,267}$ = 0.38	1.00	1.00	$F_{100,271}$ = 0.36	1.00	1.00
	Session x ToT x Day	$F_{20,271}$ = 1.14	0.31	0.56	$F_{20,265}$ = 1.06	0.40	0.66	$F_{20,254}$ = 0.90	0.59	0.81
	Quadrupled interaction	$F_{20,271}$ = 0.35	1.00	1.00	$F_{40,267}$ = 0.41	0.99	1.00	$F_{100,271}$ = 0.39	1.00	1.00
**PVT Standard deviation**	Genotype	$F_{1,63}$ = 0.64	0.43	0.69	$F_{2,62}$ = 0.08	0.92	0.98	$F_{5,59}$ = 0.50	0.78	0.91
	Session	$F_{5,713}$ = 6.90	$2.68E^{-06}$	$1.32E^{-05}$	$F_{5,727}$ = 8.17	$1.62E^{-07}$	$9.86E^{-07}$	$F_{5,649}$ = 7.03	$2.05E^{-06}$	$1.06E^{-05}$
	ToT	$F_{4,826}$ = 2.27	$6.00E^{-02}$	0.14	$F_{4,851}$ = 2.24	$6.29E^{-02}$	0.14	$F_{4,770}$ = 2.31	$5.61E^{-02}$	0.13
	Day	$F_{1,2080}$ = 258.25	$7.21E^{-55}$	$2.44E^{-53}$	$F_{1,2122}$ = 233.64	$4.05E^{-50}$	$1.20E^{-48}$	$F_{1,1971}$ = 219.79	$3.20E^{-47}$	$8.41E^{-46}$
	Genotype x Session	$F_{5,713}$ = 2.16	$5.68E^{-02}$	0.14	$F_{10,724}$ = 1.77	$6.29E^{-02}$	0.14	$F_{25,669}$ = 1.72	$1.69E^{-02}$	$4.70E^{-2}$
	Genotype x ToT	$F_{4,826}$ = 0.95	0.44	0.70	$F_{8,845}$ = 0.18	0.99	1.00	$F_{20,769}$ = 0.55	0.94	0.99
	Genotype x Day	$F_{1,2080}$ = 0.37	0.54	0.78	$F_{2,2106}$ = 0.74	0.48	0.74	$F_{5,1965}$ = 0.77	0.57	0.80
	Session x ToT	$F_{20,397}$ = 0.81	0.71	0.90	$F_{20,379}$ = 0.83	0.68	0.88	$F_{20,441}$ = 0.76	0.76	0.91
	Session x Day	$F_{5,713}$ = 2.47	$3.14E^{-02}$	$7.99E^{-02}$	$F_{5,727}$ = 1.96	$8.30E^{-02}$	0.19	$F_{5,649}$ = 2.15	$5.78E^{-02}$	0.14
	ToT x Day	$F_{4,826}$ = 0.86	0.49	0.74	$F_{4,851}$ = 0.73	0.58	0.80	$F_{4,770}$ = 0.53	0.72	0.90
	Genotype x ToT x Day	$F_{4,826}$ = 1.96	$9.85E^{-02}$	0.22	$F_{8,845}$ = 1.43	0.18	0.37	$F_{20,769}$ = 1.18	0.27	0.49
	Genotype x Session x Day	$F_{5,713}$ = 2.87	$1.41E^{-02}$	$3.99E^{-02}$	$F_{10,724}$ = 1.61	$9.88E^{-02}$	0.22	$F_{25,669}$ = 1.91	$5.15E^{-03}$	$1.61E^{-2}$
	Genotype x Session x ToT	$F_{20,397}$ = 0.70	0.83	0.94	$F_{40,387}$ = 1.00	0.47	0.73	$F_{100,436}$ = 1.07	0.32	0.57
	Session x ToT x Day	$F_{20,397}$ = 1.11	0.33	0.58	$F_{20,379}$ = 1.23	0.22	0.44	$F_{20,414}$ = 1.22	0.23	0.44
	Quadrupled interaction	$F_{20,397}$ = 0.580	0.93	0.98	$F_{40,387}$ = 1.17	0.23	0.44	$F_{100,436}$ = 0.94	0.65	0.86
**Subjective sleepiness**	Genotype	$F_{1,80}$ = 0.59	0.45	0.70	$F_{2,78}$ = 0.73	0.49	0.74	$F_{5,74}$ = 1.45	0.22	0.42
	Session	$F_{5,553}$ = 17.24	$7.24E^{-16}$	$6.60E^{-15}$	$F_{5,439}$ = 5.22	$4.08E^{-04}$	$1.46E^{-03}$	$F_{5,507}$ = 15.75	$1.99E^{-14}$	$1.63E^{-13}$

	Day	$F_{1,213}$ = 884.54	9.42E^{-78}	5.58E^{-76}	$F_{1,161}$ = 587.12	1.66E^{-55}	6.55E^{-54}	$F_{1,201}$ = 756.34	5.35E^{-70}	2.54E^{-68}
	Genotype x Session	$F_{5,553}$ = 0.32	0.90	0.97	$F_{10,439}$ = 0.63	0.75	0.91	$F_{25,508}$ = 0.58	0.95	1.00
	Genotype x Day	$F_{1,213}$ = 0.47	0.49	0.74	$F_{2,161}$ = 0.63	0.88	0.97	$F_{5,201}$ = 3.51	4.59E^{-03}	1.45E^{-02}
	Session x Day	$F_{5,541}$ = 3.29	6.12E^{-03}	1.88E^{-02}	$F_{5,453}$ = 3.04	1.71E^{-02}	4.72E^{-02}	$F_{5,502}$ = 2.52	2.87E^{-02}	7.39E^{-02}
	Genotype x Session x Day	$F_{5,541}$ = 0.16	0.98	1.00	$F_{10,454}$ = 0.44	0.90	0.97	$F_{25,502}$ = 0.48	0.99	1.00
**Waking EEG Delta band 1 – 4.5 Hz**	Genotype	$F_{1,79}$ = 0.60	0.44	0.70	$F_{2,77}$ = 1.24	0.30	0.54	$F_{5,74}$ = 0.64	0.67	0.88
	Day	$F_{1,209}$ = 108.42	1.01E^{-20}	1.33E^{-19}	$F_{1,201}$ = 98.40	3.88E^{-19}	4.38E^{-18}	$F_{1,193}$ = 100.88	2.32E^{-19}	2.75E^{-18}
	Session	$F_{5,612}$ = 7.08	1.90E^{-06}	1.00E^{-05}	$F_{5,592}$ = 6.03	1.85E^{-05}	7.54E^{-05}	$F_{5,568}$ = 6.08	1.68E^{-05}	6.98E^{-05}
	Genotype x Day	$F_{1,209}$ = 0.13	0.72	0.90	$F_{2,202}$ = 0.34	0.71	0.90	$F_{5,193}$ = 0.85	0.52	0.75
	Genotype x Session	$F_{5,612}$ = 1.00	0.42	0.69	$F_{10,593}$ = 1.09	0.37	0.63	$F_{25,573}$ = 0.97	0.50	0.75
	Session x Day	$F_{5,565}$ = 5.03	1.61E^{-04}	6.06E^{-04}	$F_{5,547}$ = 4.21	9.23E^{-04}	3.17E^{-03}	$F_{5,525}$ = 4.47	5.31E^{-04}	1.85E^{-03}
	Genotype x Session x Day	$F_{5,565}$ = 1.45	0.21	0.41	$F_{10,547}$ = 0.54	0.86	0.96	$F_{25,539}$ = 0.80	0.75	0.91
**Waking EEG Theta band 5 – 7.5 Hz**	Genotype	$F_{1,79}$ = 0.04	0.85	0.95	$F_{2,77}$ = 2.24	0.11	0.24	$F_{5,74}$ = 1.20	0.32	0.57
	Day	$F_{1,191}$ = 233.03	6.26E^{-35}	1.48E^{-33}	$F_{1,185}$ = 211.86	1.69E^{-32}	3.33E^{-31}	$F_{1,180}$ = 221.20	3.95E^{-33}	8.51E^{-32}
	Session	$F_{5,619}$= 8.26	1.46E^{-07}	9.08E^{-07}	$F_{5,601}$ = 6.51	6.54E^{-06}	2.87E^{-05}	$F_{5,575}$ = 6.08	1.67E^{-05}	6.98E^{-05}
	Genotype x Day	$F_{1,191}$ = 0.18	0.68	0.88	$F_{2,186}$ = 1.13	0.33	0.57	$F_{5,180}$ = 1.96	8.68E^{-02}	0.19
	Genotype x Session	$F_{5,619}$ = 0.33	0.90	0.97	$F_{10,602}$ = 0.88	0.56	0.80	$F_{25,577}$ = 0.88	0.64	0.86
	Session x Day	$F_{5,559}$ = 9.17	2.15E^{-08}	1.38E^{-07}	$F_{5,543}$ = 6.89	3.01E^{-06}	1.49E^{-05}	$F_{5,521}$ = 6.77	3.95E^{-06}	1.87E^{-05}
	Genotype x Session x Day	$F_{5,559}$ = 0.91	0.47	0.73	$F_{10,544}$ = 1.35	0.20	0.40	$F_{25,523}$ = 1.09	0.35	0.60
**Waking EEG Alpha band 8 – 11.5 Hz**	Genotype	$F_{1,79}$ = 0.00	0.97	1.00	$F_{2,77}$ = 1.05	0.36	0.61	$F_{5,74}$ = 0.79	0.56	0.80
	Day	$F_{1,171}$ = 2.06	0.15	0.32	$F_{1,163}$ = 5.45	2.08E^{-02}	5.53E^{-02}	$F_{1,162}$ = 4.42	3.71E^{-02}	9.26E^{-02}
	Session	$F_{5,638}$ = 9.39	1.19E^{-08}	8.09E^{-08}	$F_{5,622}$ = 7.96	2.78E^{-07}	1.65E^{-06}	$F_{5,594}$ = 7.86	3.52E^{-07}	2.03E^{-06}
	Genotype x Day	$F_{1,171}$ = 10.65	1.33E^{-03}	4.49E^{-03}	$F_{2,164}$ = 3.01	5.20E^{-02}	0.13	$F_{5,162}$ = 3.97	2.02E^{-03}	6.75E^{-03}
	Genotype x Session	$F_{5,638}$ = 0.62	0.69	0.89	$F_{10,622}$ = 0.66	0.77	0.91	$F_{25,601}$ = 0.76	0.79	0.91
	Session x Day	$F_{5,561}$ = 5.97	2.15E^{-05}	8.62E^{-05}	$F_{5,546}$ = 5.40	7.33E^{-05}	2.85E^{-04}	$F_{5,524}$ = 4.88	2.26E^{-04}	8.37E^{-04}
	Genotype x Session x Day	$F_{5,561}$ = 0.59	0.71	0.90	$F_{10,546}$ = 0.76	0.67	0.88	$F_{25,528}$ = 0.75	0.81	0.92
**Waking EEG Theta/Alpha ratio**	Genotype	$F_{1,79}$ = 0.02	0.89	0.97	$F_{2,77}$ = 0.54	0.59	0.81	$F_{5,74}$ = 0.78	0.57	0.80
	Day	$F_{1,166}$ = 187.57	4.47E^{-29}	7.06E^{-28}	$F_{1,160}$ = 196.91	1.10E^{-29}	1.85E^{-28}	$F_{1,158}$ = 202.13	4.62E^{-30}	8.42E^{-29}
	Session	$F_{5,629}$ = 2.19	5.36E^{-02}	0.13	$F_{5,608}$ = 1.85	0.10	0.22	$F_{5,580}$ = 1.79	0.11	0.24
	Genotype x Day	$F_{1,166}$ = 8.58	3.88E^{-03}	1.26E^{-02}	$F_{2,160}$ = 6.14	2.69E^{-03}	8.85E^{-03}	$F_{5,158}$ = 5.03	2.62E^{-04}	9.55E^{-04}
	Genotype x Session	$F_{5,626}$ = 0.74	0.59	0.81	$F_{10,609}$ = 1.97	3.45E^{-02}	8.70E^{-02}	$F_{25,584}$ = 1.17	0.26	0.48
	Session x Day	$F_{5,549}$ = 1.72	0.13	0.27	$F_{5,533}$ = 2.26	4.76E^{-02}	0.12	$F_{5,512}$ = 1.79	0.11	0.24
	Genotype x Session x Day	$F_{5,549}$ = 0.45	0.81	0.92	$F_{10,533}$ = 1.34	0.21	0.41	$F_{5,515}$ = 1.07	0.38	0.64
**Waking EEG Low Beta 12 – 19.5 Hz**	Genotype	$F_{1,79}$ = 0.43	0.51	0.75	$F_{2,77}$ = 0.57	0.57	0.80	$F_{5,74}$ = 0.88	0.50	0.75
	Day	$F_{1,205}$ = 24.43	1.60E^{-06}	8.61E^{-06}	$F_{1,198}$ = 22.37	4.26E^{-06}	1.98E^{-05}	$F_{1,191}$ = 22.72	3.69E^{-06}	1.79E^{-05}
	Session	$F_{5,590}$ = 13.63	1.33E^{-12}	1.05E^{-11}	$F_{5,573}$ = 11.60	1.09E^{-10}	8.06E^{-10}	$F_{5,552}$ = 10.50	1.21E^{-09}	8.70E^{-09}

	Genotype x Day	$F_{1,205}$ = 1.77	0.19	0.38	$F_{2,199}$ = 0.68	0.51	0.75	$F_{5,192}$ = 0.87	0.50	0.75
	Genotype x Session	$F_{5,590}$ = 1.18	0.32	0.57	$F_{10,574}$ = 0.68	0.74	0.91	$F_{25,557}$ = 0.80	0.74	0.91
	Session x Day	$F_{5,549}$ = 20.74	$5.26E^{-19}$	$5.67E^{-18}$	$F_{5,533}$ = 17.42	$5.50E^{-16}$	$5.21E^{-15}$	$F_{5,513}$ = 17.09	$1.20E^{-15}$	$1.05E^{-14}$
	Genotype x Session x Day	$F_{5,549}$ = 0.65	0.66	0.88	$F_{10,534}$ = 0.51	0.88	0.97	$F_{25,517}$ = 0.49	0.98	1.00
**Waking EEG High Beta 20 – 30 Hz**	Genotype	$F_{1,79}$ = 0.61	0.44	0.70	$F_{2,77}$ = 0.44	0.65	0.86	$F_{5,74}$ = 1.04	0.40	0.66
	Day	$F_{1,178}$ = 5.65	$1.85E^{-02}$	$4.98E^{-02}$	$F_{1,172}$ = 6.88	$9.49E^{-03}$	$2.78E^{-02}$	$F_{1,165}$ = 6.57	$1.13E^{-02}$	$3.25E^{-02}$
	Session	$F_{5,582}$ = 3.43	$4.58E^{-03}$	$1.45E^{-02}$	$F_{5,568}$ = 2.94	$1.25E^{-02}$	$3.56E^{-02}$	$F_{5,547}$ = 2.76	$1.79E^{-02}$	$4.87E^{-02}$
	Genotype x Day	$F_{1,178}$ = 0.79	0.38	0.64	$F_{2,172}$ = 0.90	0.41	0.67	$F_{5,154}$ = 0.47	0.80	0.91
	Genotype x Session	$F_{5,582}$ = 0.32	0.90	0.97	$F_{10,569}$ = 1.20	0.29	0.54	$F_{25,551}$ = 0.78	0.78	0.91
	Session x Day	$F_{5,529}$ = 6.69	$4.64E^{-06}$	$2.11E^{-05}$	$F_{5,515}$ = 5.74	$3.65E^{-05}$	$1.44E^{-04}$	$F_{5,495}$ = 5.23	$1.08E^{-04}$	$4.13E^{-04}$
	Genotype x Session x Day	$F_{5,529}$ = 0.53	0.76	0.91	$F_{10,516}$ = 1.29	0.23	0.44	$F_{25,497}$ = 0.87	0.65	0.86

q-Values: FDR-corrected p-values. Note: The FRD correction is performed across all three genetic groups and therefore takes into account all 237 p-values shown in the table. Subscript F values represent numerator and denominator degrees of freedom, respectively. Red values are below the standard 5% alpha criteria.
